# Amazonian terrestrial water balance inferred from satellite-observed water vapor isotopes

**DOI:** 10.1038/s41467-022-30317-4

**Published:** 2022-05-13

**Authors:** Mingjie Shi, John R. Worden, Adriana Bailey, David Noone, Camille Risi, Rong Fu, Sarah Worden, Robert Herman, Vivienne Payne, Thomas Pagano, Kevin Bowman, A. Anthony Bloom, Sassan Saatchi, Junjie Liu, Joshua B. Fisher

**Affiliations:** 1 Joint Institute for Regional Earth System Science and Engineering, University of California, Los Angeles, CA USA; 2 Pacific Northwest National Laboratory, 902 Battelle Blvd, Richland, WA USA; 3 Jet Propulsion Laboratory, California Institute of Technology, Pasadena, CA USA; 4 National Center for Atmospheric Research, Boulder, CO USA; 5 University of Auckland, Auckland, New Zealand; 6 Laboratoire de Météorologie Dynamique, Paris, France; 7 University of California, Los Angeles, CA USA; 8 California Institute of Technology, Pasadena, CA USA; 9 Schmid College of Science and Technology, Chapman University, 1 University Drive, Orange, CA USA

**Keywords:** Atmospheric chemistry, Hydrology

## Abstract

Atmospheric humidity and soil moisture in the Amazon forest are tightly coupled to the region’s water balance, or the difference between two moisture fluxes, evapotranspiration minus precipitation (ET-P). However, large and poorly characterized uncertainties in both fluxes, and in their difference, make it challenging to evaluate spatiotemporal variations of water balance and its dependence on ET or P. Here, we show that satellite observations of the HDO/H_2_O ratio of water vapor are sensitive to spatiotemporal variations of ET-P over the Amazon. When calibrated by basin-scale and mass-balance estimates of ET-P derived from terrestrial water storage and river discharge measurements, the isotopic data demonstrate that rainfall controls wet Amazon water balance variability, but ET becomes important in regulating water balance and its variability in the dry Amazon. Changes in the drivers of ET, such as above ground biomass, could therefore have a larger impact on soil moisture and humidity in the dry (southern and eastern) Amazon relative to the wet Amazon.

## Abstract (web summary)

The evolution of the Amazon forest is tightly coupled to its terrestrial water balance. Here, the authors show that forest biomass changes in the Amazon are a driver of the spatiotemporal variation of evapotranspiration, and such changes could have a larger impact on water availability in the dry regions (southern, eastern) of the Amazon.

## Introduction

The Amazon biome typically receives rainfall exceeding ~2000 mm year^−1^ ^1^. This rainfall depends on advected oceanic moisture combined with (ET). ET can contribute up to 30–40% of the atmospheric moisture during the dry season^2–4^ and is important for the initiation of the seasonal monsoon over the southern Amazon^5^. Characterizing the moisture balance between ET and precipitation (or ET-P) and how it is affected by climate and anthropogenic forcings is therefore critical for evaluating the variability of forest dynamics (e.g., carbon storage, forest structure, and composition) in both the wet Amazon and the surrounding dry tropics. For example, an initial increase in ET-P over the wet Amazon represents a local loss in soil moisture but an increase in atmospheric humidity. This humidity becomes a source of rainfall for local and downwind forests^3^, which helps to maintain photosynthesis and forest growth in the dry tropics^1,6^. However, a continuous local water balance deficiency (i.e., a long-term increase in ET-P), could induce forest biomass reduction in these areas with soil moisture loss^7–9^.

Because ET-P is equivalent to the change in water storage and river discharge, it can be quantified using satellite gravity-based terrestrial water storage (TWS) and river discharge measurements^10–12^ (“Methods”). However, limited spatial resolution of the gravity measurements and limited accuracy of the river measurements mean that these water balance estimates have very coarse spatial resolution of about 800 × 800 km^2^ (i.e., river-basin scale)^10^. Furthermore, they can only be derived in the wet Amazon, where discharge measurements are representative of river basins of similar spatial resolution to the gravity measurements. Water balance can also be quantified using precipitation and ET products from remote sensing or reanalysis. However, quantifying the uncertainties of these products can also be challenging^13^, since their accuracies and precision vary with cloud cover^14,15^ and rainfall.

**Fig. 1:**
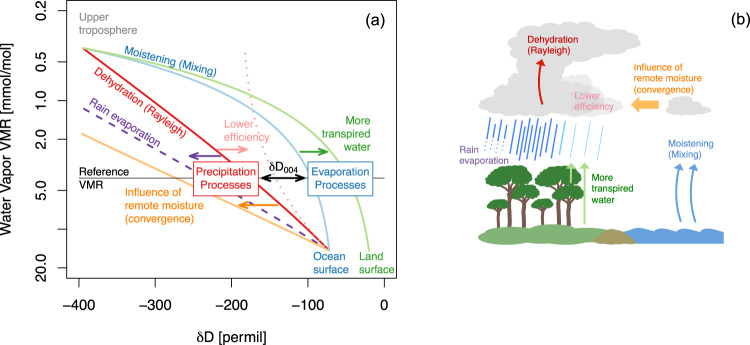
The diagram of water vapor and δ*D* (δ*D*_004) dynamics. **a** The processes influencing δ*D*_004 variability, shown on a plot of water vapor volume mixing ratio (*y*-axis) versus δ*D* (*x*-axis). For a constant water vapor volume mixing ratio (4 mmol mol^−1^, flat gray line labeled “Reference VMR”), variations in the hydrogen isotope ratio (δ*D*_004, shown in the Figure as δ*D*_004_) represent the shifting importance of precipitation vs. evapotranspiration (ET-P). The precise scaling of δ*D*_004 to ET-P will be modified by the efficiency of rainout (i.e., the efficiency with which cloud condensate is converted to rain) and by the source of moisture to the atmosphere. For example, the two-sided black arrow shows the expected range of δ*D*_004 if oceanic evaporation is the sole source of moisture to the atmosphere (blue line) and condensate formed during convection is immediately removed from the atmosphere by precipitation (red line). The intersection of the blue and red lines with the “Reference VMR” line explicitly shows the expected δ*D*_004 values if *P* = 0 or ET = 0, respectively. As the contribution of transpiration to atmospheric moistening increases (green line), the δ*D*_004 range will extend to the right, causing the expected δ*D*_004 value to be higher when *P* = 0. Contrastingly, as either rain evaporation (purple dashed line) or remote moisture convergence (orange line) becomes important, the δ*D*_004 range will extend to lower isotope ratios, causing the expected δ*D*_004 value to be lower when ET = 0. Decreasing the efficiency with which condensate forms precipitation (pink dotted line) will, in comparison, increase the expected δ*D*_004 value when ET = 0, limiting the expected δ*D*_004 range for a given set of ET-P states. **b** The schematic illustrates the key processes in (**a**).

Here, we describe the relationship between the deuterium content of water vapor (in this case the number of HDO molecules to the number of H_2_O molecules or HDO/H_2_O ratio) and its relationship to ET and precipitation. Satellite observations of HDO/H_2_O ratio have been used for evaluating the primary moisture sources and dynamics affecting the tropical and sub-tropical water cycle^5,16–21^. The deuterium content of a measurement is traditionally given in parts per thousand relative to the deuterium content of ocean water (i.e., δ*D*; “Methods”). Consequently, a value of zero means the measurement has the same deuterium content as the ocean and a value of −1000 permil means the measured air parcel has no deuterium. Precipitation and mixing processes change the atmosphere’s deuterium content and its bulk moisture content; thus, δ*D* covaries, to zeroth order, with specific humidity. However, the exact nature of this covariance depends on which precipitation or mixing process is dominant (Fig. 1)^17,22^. During a rainfall event, the deuterium content is gradually depleted, broadly following what is called a Rayleigh distillation^23^. In contrast, when the free troposphere mixes with evapo-transpired water vapor in the boundary layer, the deuterium content increases^5^. Furthermore, since transpiration and the complete evaporation of intercepted water from forest canopies produce little-to-no net fractionation, water vapor sourced from heavily vegetated areas is typically more enriched than water vapor originating from the ocean^17,21,24^.

The degree to which isotopic variations follow one or the other of these precipitation or evaporation relationships is readily quantified by normalizing the δ*D* to a reference water vapor concentration. This normalization removes the dependence of the HDO/H_2_O ratio on H_2_O (Fig. 1a). Comparing observations to mixing models can then provide an additional constraint on the origin of the observed vapor and its variability^21,22,25,26^. Research using model simulations^27^ has found that normalizing the observed HDO/H_2_O ratio in vapor to a reference value of 0.004 volume mixing ratio (VMR), typical of free-tropospheric concentrations (“Methods”), creates a linear proxy for ET-P^27,28^ over the tropical ocean. We call this proxy δ*D*_004 and use it to quantify water balance as discussed next.

In this work, we show that satellite measurements of HDO/H_2_O ratio of water vapor reflects the spatial, seasonal, and interannual variability (IAV) of ET-P over the Amazon, as opposed to either flux alone, mitigating uncertainty related to taking the difference of two very large fluxes. These isotopic measurements are derived from thermal infrared radiances measured by the Atmospheric Infrared Sounder (AIRS) instrument. The AIRS measurements span 2002 through the present and are accurately calibrated over this time period relative to ground and aircraft data^29^, making them suitable for quantifying the HDO/H_2_O ratio of tropospheric vapor from seasonal to decadal time scales. We calibrate the isotopic data to ET-P at river-basin scales using satellite gravity-based TWS and river discharge (herein TWS/discharge) measurements (“Methods”), and quantify their spatial biases and precision using a global climate model enabled with water isotopes. We then quantify the relationship between precipitation and water balance in the wet and dry Amazon in order to evaluate if ET or precipitation variability is the primary factor controlling seasonal water balance variability.

## Results

### Quantifying ET-P and its uncertainties from AIRS deuterium measurement

**Fig. 2:**
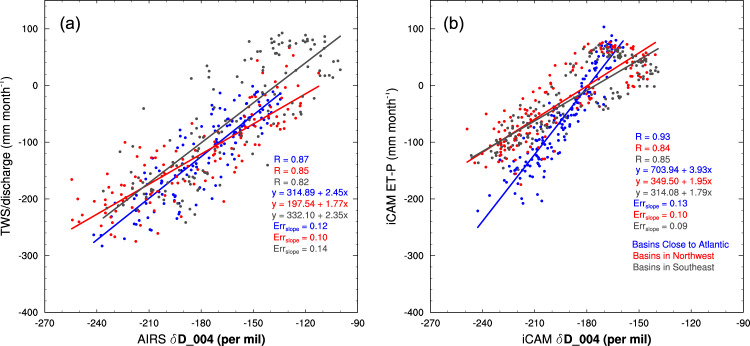
The regressions of ET-P on δD_004 from both observations and iCAM. **a** represents the regressions of TWS/discharge on AIRS δD_004 and **b** represents the regressions of ET-P on δD_004 from iCAM during 2013–2015. Here, we use observations and iCAM output at a monthly time scale. Basins close to the Atlantic are river basins 1, 3, 10, and 12 from Fig. 3b; basins in the Northwest are basins 5, 8, 9,13, and 14; basins in the Southeast are basins 2, 4, 6, 7, and 11. For each region, we calculate the area-weighted average of ET-P from TWS/discharge, AIRS δ*D*_004, and ET-P and δD_004 from iCAM for all months during 2003–2015. Here, the region comprised of basins close to the Atlantic has a higher slope than the other regions, according to both the observations and iCAM, indicating a different moisture source.

We use monthly gridded AIRS deuterium measurements during 2003–2015 (“Methods”) and corresponding δ*D*_004 estimates (“Methods”) to analyze seasonal, interannual, and spatial variability in ET-P for the Amazon (Table S1) and surrounding regions. The measurements have a monthly uncertainty of ~4 permil with no observable changes in the calibration over the observational time period^30^, making the record suitable for evaluating seasonal to decadal changes in the tropospheric HDO/H_2_O ratio. Figure 2a demonstrates how the AIRS δ*D*_004 varies with ET-P, estimated from TWS and river discharge data. We show these relationships for three regions: the northeastern, southeastern, and western parts of the Amazon, which are large enough to be resolved by TWS retrievals from the Gravity Recovery and Climate Experiment (GRACE) observations. Because advection from the Atlantic Ocean during the rainy season typically enters the Amazon from the northeast and then circulates along the Andes towards the south^31^, we also use these regions to evaluate how changes in moisture sources affect the δ*D*_004 versus ET-P relationship.

The comparisons demonstrate linearity between the ET-P estimates from TWS/discharge and AIRS δ*D*_004. Differences in the slope and offset between the three regions are likely due to the fact that areas nearer the ocean are more dependent on the less enriched ocean moisture source while areas farther inland have a larger ET signal with correspondingly higher deuterium content^21^. Based on these results, we conclude that we can use river-basin-scale comparisons to quantify ET-P from δ*D*_004 (“Methods”), so long as we account for spatial variations in the regression coefficients that project δ*D*_004 to ET-P across the Amazon.

### Quantifying uncertainty of ET-P variability based on δ*D*_004 using the isotope enabled Community Atmosphere Model

We use the isotope enabled Community Atmosphere Model Version 5 (herein iCAM; “Methods”) to (1) further demonstrate that we expect a linear relationship between δ*D*_004 and ET-P and (2) quantify uncertainties in our method of using regressions of TWS/discharge and AIRS δD_004 at river-basin scales to estimate ET-P across the Amazon. Figure 2b shows ET-P versus δ*D*_004 in iCAM for the same regions shown in Fig. 2a. As with the observed relationships between AIRS δ*D*_004 and TWS/discharge, the modeled relationships are linear (correlation coefficients of 0.84 or higher) for the wet Amazon. However, observed ET-P is largely negative throughout the year over the wet tropics (i.e., in river basins close to the Atlantic and in northwestern Amazon), whereas iCAM has ET-P being positive for ~1/3 of the year. Because of these large differences, we cannot use iCAM as a way to calibrate the δ*D*_004 proxy. Instead, we use the iCAM model to evalute uncertainties in the proxy and whether seasonal changes in dynamics and moisture sources change the ET-P and δ*D*_004 relationship. We quantify this uncertainty as the root-mean square (RMS) difference between the iCAM ET-P simulation and the ET-P derived from the ET-P and δ*D*_004 relationships from iCAM (i.e., the residual standard deviation of the regressions shown in Fig. 2b). Because the uncertainty in the AIRS deuterium data is relatively small for monthly averages (~4 per mil)^30^, the primary source of scatter in Fig. 2b is likely the variable sources of ET and precipitation or the isotopic physics used in iCAM. However, other processes not well modeled by iCAM could also affect the scatter; these include variations in cloud microphysical processes and changes in the depth of convection resulting in variations in moisture flux convergence (Fig. 1). All these processes are discussed in the “Methods” and are shown to have a negligible impact on the precision of the deuterium-based ET-P estimates from AIRS. The scatter shown in Fig. 2b represents the uncertainty on the seasonal variability of the δ*D*_004-based ET-P estimates. The overall accuracy is bounded by the TWS/discharge measurements as discussed next.

### Accuracy of ET-P Estimates

**Fig. 3:**
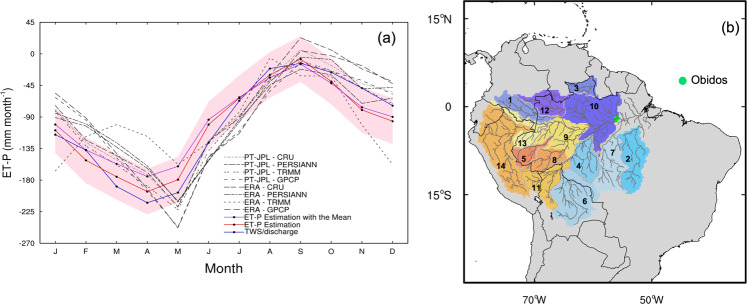
The seasonality of evapotranspiration minus precipitation (ET-P) in one of the Amazon sub-basin groups and the Amazon basin distribution. **a** The seasonality of ET-P is from different ET and P data sources, TWS/discharge, and ET-P estimates based on AIRS δ*D*_004 for the geographic area composed of river basins 3, 10, and 12 (Group 1; “Methods”) during 2003–2015. Panel **b** shows the river basin distribution map for the Amazon. In **a**, we use area-weighted averaging. We use two ET sources; one is from PT-JPL ET, the other is the latent heat flux of ERA5. We also use four precipitation products: TRMM, GPCP, PERSIANN, and CRU. Other monthly data products include terrestrial water storage (TWS) from GRACE, river discharge from Amazon river gauge measurements, and ET-P derived from AIRS δ*D*_004. “ET-P Estimation” is the ET-P estimate from AIRS δ*D*_004 based on the regression against TWS/discharge. “ET-P Estimation with Mean” is an alternative estimate of ET-P from AIRS δ*D*_004 based on regressing the isotopic data against the average ET-P derived from the eight combinations of remote-sensing and reanalysis moist flux products. The pink shading represents the suggested error in the fit between monthly ET-P and δ*D*_004 from iCAM.

We estimate the accuracy of the ET-P estimates based on the AIRS δ*D*_004 data as the RMS error in the fit between the monthly based TWS/discharge versus AIRS δ*D*_004 estimates of ET-P and TWS/discharge suggested ET-P. We use TWS/discharge for this purpose because GRACE TWS is precision limited at river-basin scales and its uncertainties have been quantified in the literature^10^. The accuracy is shown in Table 1 for five groups of river basins described in the “Methods”. We assess the accuracy through comparisons with different ET-P esimates derived from different ET and precipitation remote sensing and reanalysis products (“Methods”; Table S2). Results from Group 1, composed of river basins 3, 10, and 12 (which are shown in Fig. 3b and named in Table S1), are discussed as an example, because these three basins do not have any missing values for the river discharge measurements during 2003–2015. We find that the RMS errors in the fit between the ET-P estimates derived from different moisture flux products and AIRS δ*D*_004 are generally larger but within a factor of two of the RMS errors in the fit derived from TWS/discharge and AIRS δ*D*_004 (Table S2). This suggests that our choice of using TWS/discharge data to calibrate the AIRS δ*D*_004 proxy and our estimate of its accuracy are reasonable.

**Table 1:** The regression coefficient and standard error of the regression coefficient, intercept, correlation coefficient, and the root-mean square (RMS) error in the fit (mm month^−1^) between ET-P and δ*D*_004 in the 5 river basin groups: Group 1 (basins 3, 10 and 12), Group 2 (basins 1, 13, and 14), Group 3 (basins 5, 8, and 9), Group 4 (basins 4, 6, and 11), and Group 5 (basins 2 and 7).

Group number	Regression coefficient ± standard error of the regression coefficient	Intercept	Correlation coefficient	Error in the fit (mm month^−1^)
1	2.62 ± 0.13	353.60 ± 23.06	0.85	43.11
2	1.66 ± 0.09	172.67 ± 17.60	0.84	35.86
3	1.91 ± 0.12	239.62 ± 21.86	0.82	54.86
4	1.91 ± 0.12	258.93 ± 19.99	0.80	52.89
5	2.86 ± 0.20	383.40 ± 32.08	0.78	77.85

The calculations are based on ET-P estimated with GRACE TWS and river discharge (TWS/discharge; mm month^−1^) and δ*D*_004 (per mil) from AIRS during 2003–2015.

### Seasonality of ET-P based on δ*D*_004 measurements

Figure 3a shows the monthly average AIRS δ*D*_004-based ET-P estimates from Group 1, which is composed of river basins 3, 10, and 12 (Fig. 3b and Table S1). We compare these estimates to (1) ET-P from TWS/discharge, (2) eight other ET-P estimates calculated from the same moisture flux products used for assessing accuracy in Table S2, (3) an ET-P estimate using the regression coefficients from the TWS/discharge–δ*D*_004 comparison, and (4) a similar ET-P estimates using AIRS δ*D*_004 versus the mean of the eight other ET-P calculations from Table S2. The pink shading describes the uncertainty in the deuterium-based ET-P variability; as discussed earlier, this uncertainty is calculated using the iCAM simulations. Across all five groups of river basins (defined in the “Methods”), the uncertainties range from ~32 to 41 mm month^−1^ (Table 2). We find that the seasonality of the deuterium-based ET-P estimates agrees best with the ET-P obtained from TWS/discharge; however, noticeable differences are seen in the early and late part of the wet season (December and May). The comparisons demonstrate that the deuterium-based ET-P estimates can resolve monthly variations in ET-P^26–28^.

**Table 2:** The regression coefficient and standard error of the regression coefficient, intercept, correlation coefficient, and the RMS error the in fit (mm month^−1^) between ET-P simulations and ET-P estimates based on simulated δ*D*_004 for the same five 5 groups of Table 1.

Group number	Regression coefficient ± standard error of the regression coefficient	Intercept	Correlation coefficient	Error in the fit (mm month^−1^)
1	3.78 ± 0.12	698.69 ± 24.49	0.91	32.60
2	2.08 ± 0.09	359.90 ± 18.14	0.85	32.99
3	1.61 ± 0.07	292.95 ± 14.56	0.84	31.99
4	1.63 ± 0.09	280.95 ± 16.24	0.81	36.57
5	2.26 ± 0.11	401.71 ± 20.47	0.84	41.41

All the calculations use ET-P (mm month^−1^) and δ*D*_004 (per mil) from iCAM during 2003–2015.

### Spatial variability of ET-P in tropical South America for wet and dry seasons

**Fig. 4:**
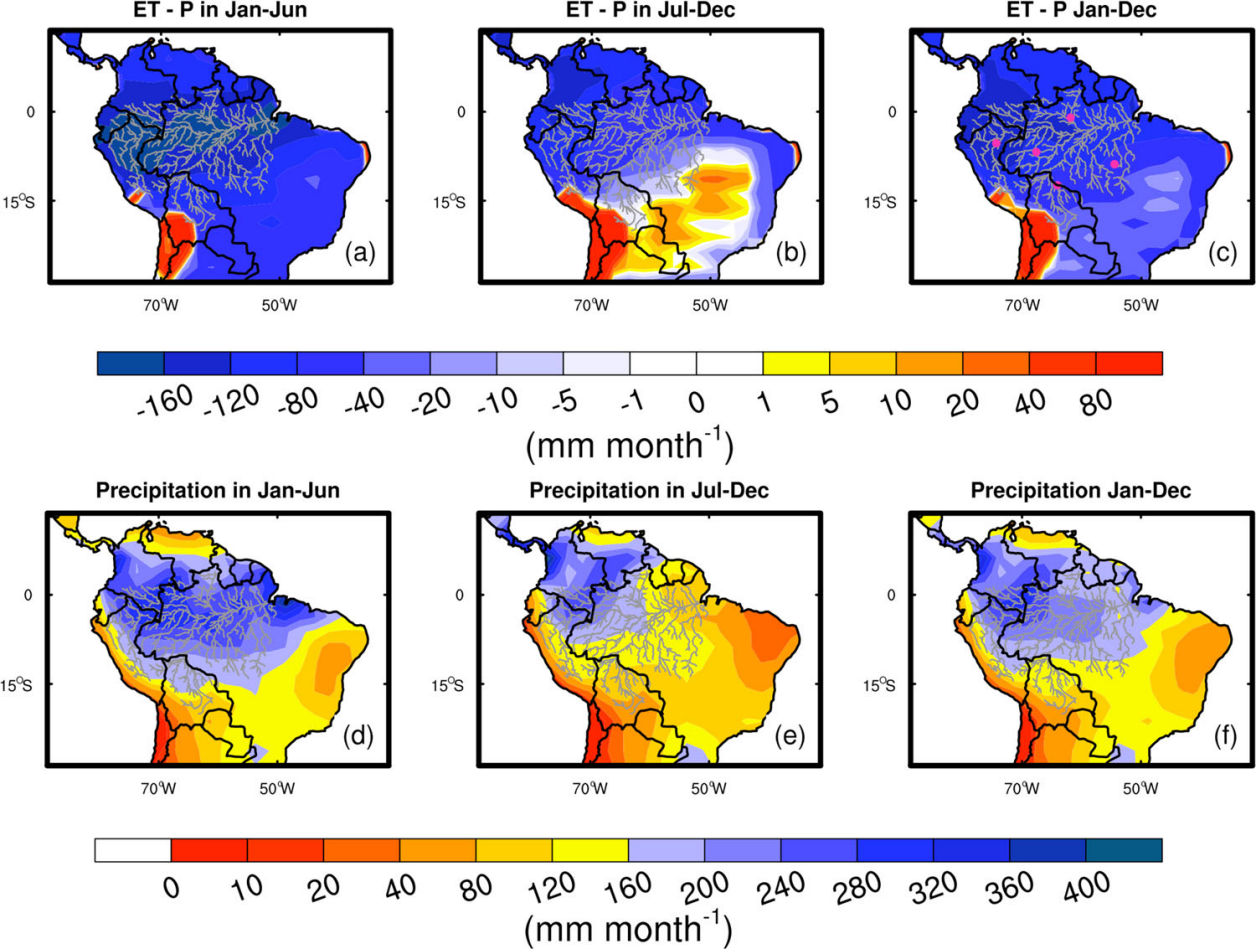
The spatial pattern of ET-P and precipitation over the Amazon. Here, we show the ET-P (mm month^−1^) derived from the TWS/discharge versus AIRS δ*D*_004 relationship for **a** the wet season (January–June), **b** the dry season (July–December), and **c** the yearly average during 2013–2019. Mean precipitation for **d** the wet season (January–June), **e** the dry season (July–December), and **f** the annual average is obtained from the monthly record of GPCP during 2013–2019. The pink dots in panel **c** indicate the center of five basin groups (“Methods”). In Figures (**d**–**f**), we use the colder colors to indicate areas with annual precipitation over 160 mm month^−1^, which is the dry-to-wet criterion of the tropics as suggested by Guan et al. (2015).

We next demonstrate how the deuterium proxy reflects the spatial as well as the seasonal variability of ET-P across the Amazon and to a lesser extent the dry tropics of South America. We first average monthly AIRS δ*D*_004 data onto a 2.5° × 2.5° grid so that it is consistent with the spatial resolution of precipitation from the Global Precipitation Climatology Project (GPCP). These gridded data are then projected to ET-P using the regression coefficients derived from comparing AIRS δ*D*_004 to TWS/discharge in the five river basin groups defined in the Methods. The regression coefficients for any given grid are calculated as a weighted mean of the basin-group-scale regression coefficients, in which the weights depend on the linear distance between the grid cell and the effective center of the river basin groups. We then use this mapped ET-P to calculate the average ET-P in the wetter months (January–June) and in the drier months (July–December) during 2003–2019 (Fig. 4). The accuracy of the estimates based on this approach might be limited in grid cells that have similar distance to a certain basin, but can avoid extrapolation errors for regions outside the Amazon basin.

Because we can apply the same mapping to the iCAM simulations, we quantify the uncertainty of extrapolating from the basin-group scale to the entire gridded Amazon. The difference between the iCAM simulations of ET-P and ET-P estimates obtained from iCAM δ*D*_004 (Table 2) shows that there is a negligible impact to the uncertainty budget from this mapping approach within the Amazon. However, deuterium-based ET-P estimates for regions outside the Amazon are likely biased low by about 40 mm month^−1^, probably because the dry troposphere sub-tropical deuterium content is correlated with that in the wet Amazon (“Methods”; Fig. S5). Consequently, care should be taken in using the deuterium-based ET-P estimates outside the Amazon (“Methods”).

## Implications and discussion

**Fig. 5:**
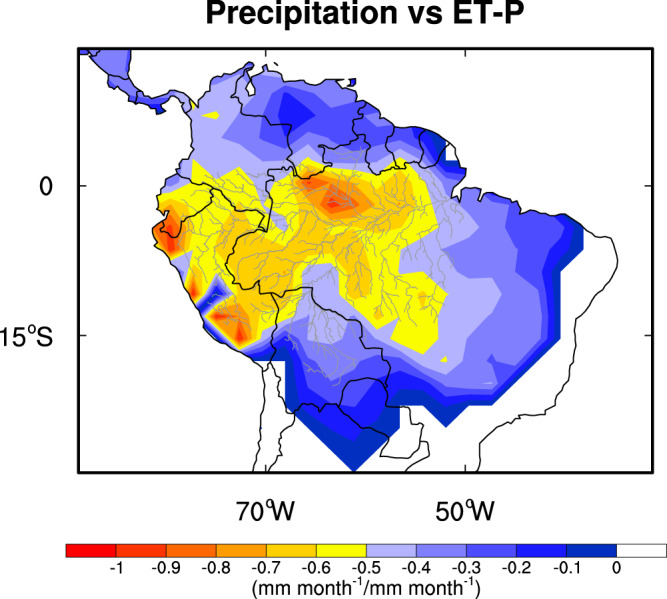
The regression slope coefficient between the ET-P (mm month^−1^) estimates based on TWS/discharge versus AIRS δ*D*_004 and GPCP precipitation (mm month^−1^) during 2003–2015. Monthly ET-P estimates and precipitation values are used for the calculation.

The deuterium-based ET-P estimates during the wetter (January–June) and drier (July–December) seasons are shown in Fig. 4a–c. Here, the dry and wet seasons are separated by the 160 mm month^−1^ precipitation criterion that is suggested by Guan et al. (2015) (the lightest blue in Fig. 4d–f). The spatial and temporal variations in Fig. 4 indicate that ET becomes more important for the water balance in the southern Amazon during the drier part of the year, especially along the arc of deforestation, which roughly aligns with the southern and eastern rivers shown in Fig. 4^32^. This result is consistent with recent research that indicates an increasing contribution of ET to atmospheric moisture for forest regions farther from the Atlantic, with the largest contribution happening during the dry season^3,5,17^. This relationship between water balance, ET, and precipitation can be further demonstrated by regressing monthly gridded water balance estimates against precipitation. Here, we regress monthly TWS/discharge versus AIRS δ*D*_004-based ET-P estimates against precipitation from the GPCP Version 2.3^33^, one of the rainfall estimates used in Fig. 3a. We then calculate the slope between these data sets for 2003–2015. We only use a single rainfall data set, since our objective is to demonstrate that the slope of ET-P versus precipitation varies from the center of the Amazon to the edges. Also, we find that GPCP is best correlated with ET-P relative to the other precipitation data sets. Regions, in which the regression slope values between ET-P and precipitation are less than −0.5, are primarily controlled by precipitation variability. If the slope value is close to zero, then ET and precipitation co-vary. Figure 5 indicates that precipitation contributes most to ET-P variability in the wet (central) Amazon but that ET becomes more important for water balance variability in the transition between the wet and dry tropics. This result confirms the importance of ET in maintaining atmospheric humidity and precipitation in this region. A caveat is that estimating the slope of ET-P versus precipitation using these data is uncertain because of noise in both of the data sets. Figure 5 thus most reliably demonstrates spatial variations between ET-P and precipitation. Based on the results shown in Figs. 4 and 5, we conclude that changes in forest structure along the arc of deforestation (roughly the region outside the southern and eastern rivers shown in Fig. 4) have an outsized effect on terrestrial water balance through changes in ET and hence are likely to affect atmospheric moisture and subsequent rainfall in this region.

While we have demonstrated that the AIRS deuterium measurements can be used to quantify the spatiotemporal Amazon water balance at monthly to seasonal and interannual time scales, further studies are needed to show that these relationships hold at decadal time scales. The AIRS calibration shows extraordinary stability^30^, and additional error reduction could also be obtained through long-term averaging. Thus, these data could be used to assess long-term changes in the water balance and determine if observed changes in climatic and anthropogenic forcings have affected the water balance over the same period. For example, interannual variations in Atlantic sea surface temperature (SST) affect drought and fire intensity^34^. Are these changes due to increasing ET-P, decreasing soil moisture, or decreasing ET-P, which reduces atmospheric humidity, or some combination of both from previous and current years (e.g., their lagged effects^35^)? Furthermore, are reductions in ET on the order of 10%^36,37^, as inferred from satellite measurements and water-budget calculations, associated with increasing water use efficiency related to CO_2_ fertilization^38^ or changes in forest structure related to fires, logging, and agriculture^11^? Given the stability of the calibration, the accuracy and precision of the data, and improved spatial resolution relative to basin-scale water balance approaches, these new deuterium-based estimates of ET-P have the potential to test one or more of these hypothetical processes controlling changes in Amazon carbon and water couplings and exchanges.

## Methods

In this section, we further explain the methods for quantifying ET-P from δ*D*_004, report the data sources and their uncertainty, and describe the iCAM configuration used in this study. Specifically, the following six sub-sections discuss (1) the method of quantifying ET-P from non-isotopic data sources, (2) the use of the tropospheric HDO/H_2_O ratio of water vapor for the estimation of ET-P, (3) sampling biases associated with the AIRS HDO/H_2_O measurements, (4) the model configuration of iCAM, (5) quantification of spatial variability and bias in the deuterium-based ET-P proxy, and (6) quantification of the uncertainty of ET-P.

### Quantifying ET-P from water balance, remote sensing of ET and P, and re-analysis

We use two independent approaches to quantify evapotranspiration minus precipitation (ET-P). One approach uses TWS retrievals from the GRACE and river discharge measurements over the Amazon by following the equation:

1 $${{{{{\rm{{ET}}}}}-P}}=\,-\frac{\varDelta W}{\varDelta t}-R,$$

where ET is evapotranspiration, P is precipitation, $\frac{\varDelta W}{\varDelta t}$ is TWS change with time, and *R* is runoff. Equation 1 is derived from the water balance equation, in which $\frac{\varDelta W}{\varDelta t}$ is estimated by subtracting ET and *R* from P^10^. Here, we use three GRACE TWS retrievals from the Center for Space Research (CSR), the GeoforschungsZentrum Potsdam (GFZ), and the Jet Propulsion Laboratory (JPL), and calculate the arithmetic mean of the three GRACE TWS retrievals^39^ to obtain $\frac{\Delta {W}}{\Delta {t}}$. During 2003–2015, there are in total 12 one-month gaps and 3 two-month gaps for GRACE TWS. We use spline interpolation to fill the gaps. The river discharge data for each river basin (Fig. 3b and Table S1) are obtained from the Observation Service for the geodynamical, Hydrological, and Biogeochemical control of erosion/alteration and material transport in the Amazon, Orinoco and Congo basins (SO-HYBAM) in-situ river-gauge discharge measurements spanning 2003–2015 (https://hybam.obs-mip.fr/). The missing records of SO-HYBAM during this time period are less than 10% of the entire time period and mostly exist at the end of the data time series; thus, the data gaps are excluded from data processing. Uncertainties are not provided with these estimates and could be as large as 20% or more for any given month^10^ based on measurements taken over well-instrumented areas in the Western USA^40^. The value for *R* in Eq. 1 is the net river discharge, that is the difference between the water leaving the basin and the water entering the basin^10,41^.

A second approach is to quantify ET-P by using independent estimates for ET and for P. Here, we use four precipitation sources: the Global Precipitation Climatology Project (GPCP) Version 2.3 at 2.5° × 2.5° and monthly *spatiotemporal* resolution^33^, the Tropical Rainfall Measuring Mission (TRMM) research product version 3B42 at 0.25° × 0.25 ° and 3-hourly *spatiotemporal* resolution^42^, the Precipitation Estimation from Remotely Sensed Information derived from Artificial Neural Networks (PERSIANN) version 1 at 0.25° × 0.25° and daily *spatiotemporal* resolution^43^, and the Climate Research Unit (CRU) version 4 at 0.5° × 0.5° and monthly *spatiotemporal* resolution^44^. Uncertainties for these precipitaiton products can range from 30% to 50% at monthly time scales^13^, depending on the amount of rain. We, therefore, take the mean of the four products and then calculate the monthly precipitation over the different river basins shown in Fig. 3b. However, this approach does not reduce biases that are a result of variable sensitivity of the radar signal to rainfall amount^45^.

We use a global ET product based on measurements from MODIS and MERRA2, utilizing the ET algorithm of Priestley Taylor-Jet Propulsion Laboratory (PT-JPL)^46^, which also forms the core global ET retrieval for NASA’s ECOSTRESS mission^47^. The uncertainty of this product for the study area is ~24 mm month^−1^ based on comparisons with surface site measurements across the tropics^48^. Recent ET estimates based on four ET algorithms (including PT-JPL) and meteorological data from 25 flux towers over South America show that the correlation between PT-JPL-estimated ET and ET obtained from measured energy balance fluxes (i.e., net radiation, soil heat flux, and sensible heat flux) is consistently higher than correlations between other model-based and surface energy balance estimates at most sites^49^. PT-JPL is also one of the algorithms with the lowest root-mean-square error (RMSE = 0.89) across the 25 South American sites. For these reasons, we use the PT-JPL ET to represent the direct remote sensing-based ET variability over the Amazon.

To estimate a monthly climatology for the years between 2003 and 2015, we calculate ET-P using TWS/discharge and ET and P products over the Obidos basin (which is comprised of all the river basins in Fig. 3b except basins 2 and 7). We choose the Obidos basin for this purpose as it covers a large area of the Amazon (i.e., 475.8 Mha). Estimated monthly mean ET-P from TWS/discharge and from ET and P products is −98 mm month^−1^ and −83 mm month^−1^, respectively. These results are consistent with previous results from both local (i.e., meteorological station)^50^ and regional^13,51^ scales, showing that ET-P is ~ −100 mm month^−1^ over the Amazon.

### Use of HDO/H_2_O ratio of free-tropospheric water vapor for quantifying ET-P

In this study, we use atmospheric deuterium measurements from the AIRS and follow the method in Bailey et al. (2017) to quantify ET-P variations. The deuterium content of a measurement is expressed in parts per thousand relative to the deuterium content of ocean waters: $\delta D=1000\left(\frac{{R}_{{{{{{\rm{obs}}}}}}}-{R}_{{{{{{\rm{std}}}}}}}}{{R}_{{{{{{\rm{std}}}}}}}}\right)$, where *R*_obs_ is the observed ratio of the number of HDO molecules to the number of H_2_^16^O molecules and *R*_std_ is the number of HDO molecules relative to the number of H_2_^16^O molecules in Vienna Standard Mean Ocean Water (*R*_std_ = 3.11 × 10^−4^).

As described extensively in previous research^17,22^, if precipitation is the sole process modifying the moisture content of the atmosphere, the deuterium content follows a well-established distillation, in which δ*D* decreases proportionally with changes in the natural logarithm of the water vapor VMR. Assuming all condensate immediately precipitates, the change in δ*D* with VMR follows the conventional Rayleigh distillation, illustrated by the red curve in Fig. 1a. In contrast, when ET is the sole process modifying the moisture content of the atmosphere, variations in δ*D* can be modeled as a simple mixing process, in which water vapor in contact with the moisture source (i.e., the ocean or land surface) mixes with and moistens the free troposphere (Fig. 1a, blue and green curves). Numerous observational studies have shown that in most regions, the vast majority of isotopic observations fall somewhere between the Rayleigh and mixing curves, reflecting the significant role that both precipitation and ET play in regulating the atmosphere’s moisture content^17,26,52,53^.

The metric δ*D*_004—first introduced in a study demonstrating that satellite measurements of water vapor isotopes can characterize variations in ET-P^27^—quantifies the degree to which the atmosphere’s hydrogen isotope ratio matches a precipitation-dominated or evaporation-dominated regime for a given water vapor concentration. Essentially, it is a measure of where the isotope ratio falls along the thin horizontal “Reference VMR” line in Fig. 1a.

To calculate δ*D*_004 for each satellite observation, the observed HDO concentration profile is linearly regressed with the collocated H_2_O concentration profile between pressure levels of approximately 325–825 hPa—an altitude region where the AIRS’ sensitivity to HDO is the largest^29^. The value of HDO for an H_2_O VMR of 0.004 is then found (herein HDO_004):

2 $${{{{{\rm{HDO}}}}}}_{004}={\beta }_{0}+{\beta }_{1}\times 0.004$$

where *β*_0_ and *β*_1_ are the intercept and slope relating the HDO and H_2_O profiles at a given time and location. The HDO_004 concentration is then converted to delta notation following:

3 $${\delta D}_{004}=\left(\frac{{{{{{{\rm{HDO}}}}}}}_{004}}{0.004\times {R}_{{{{{{\rm{std}}}}}}}}-1\right)\times 1000$$

The more ET-dominated the environment, the higher its δ*D*_004, while the more precipitation-dominated the environment, the lower its δ*D*_004. However, the exact sensitivity of δ*D*_004 to shifts in ET-P depends on the characteristics of the ET and precipitation processes in a particular region.

The double-headed black arrow in Fig. 1a represents the range of possible δ*D*_004 values for an idealized tropical environment in which the precipitating atmosphere is perfectly pseudo-adiabatic (red curve), and the marine boundary layer (with lifting condensation level dew point and δ*D* values arbitrarily set at 20 °C and −72 permil, respectively) serves as the sole moisture source. However, the δ*D*_004 range will expand or shrink (as indicated by the other arrows in Fig. 1a) depending on the characteristics of the moisture source, the efficiency of the precipitation, and the ability of convection to draw on remote moisture.

One factor that will extend the upper end of the δ*D*_004 range, for example, is the relative contribution of transpiration to the total ET flux (green arrow). Because transpiration, on balance, produces almost no isotopic fractionation, the δ*D*_004 range of tropical terrestrial environments will extend towards the green curve in Fig. 1.

The low end of the δ*D*_004 range tends to depend on the characteristics of precipitation. If, as is typical, the efficiency with which cloud water forms precipitation is less than 100%^54^, isotopic depletion during precipitation will not be as great (pink arrow). However, if rain evaporation occurs, reducing the efficiency with which precipitation reaches the surface, and if the evaporated fraction of the rain is small, the isotopic depletion will be larger (purple arrow). Importantly, evaporation has a depleting effect on the vapor only if the rain evaporated fraction is very small (first order approximation in Worden et al. 2007). Otherwise, rain evaporation has an enriching effect on the water vapor^55–57^. Microphysical processes within different regions of the atmospheric profile may thus have somewhat canceling effects on the δ*D*_004 sensitivity to ET-P.

In comparison, the convergence of remote moisture unambiguously extends the δ*D*_004 range to lower values (Fig. 1, orange arrow)^17^. Greater isotopic depletion is the result of convection incorporating ever-more rained out moisture. Indeed, idealized modeling studies indicate that the degree to which convection relies on remote moisture (as opposed to local ET) is an important factor determining tropical precipitation isotope ratios^58^. Consequently, one might expect that regions of the Amazon that lie farthest inland, whose local moisture is sourced from vegetated surfaces^3,5^, and whose non-local moisture must travel a longer distance from the Atlantic coast, are more likely to exhibit a larger δ*D*_004 sensitivity to shifts in ET-P. This suspicion is supported by the enhanced range in precipitation isotope ratios found over inland areas of the Amazon Basin (compared to coastal areas) between the precipitation-dominated wet and evaporation-dominated dry seasons^59^.

### Characterization of AIRS measurements and quantifying the clear sky sampling bias of AIRS on δ*D*_004 estimates

The deuterium content of free tropospheric water vapor used in this analysis is derived from spectral radiances measured by the NASA AIRS satellite instrument^29^. The current record spans the time period 2002 through the present. The precision of a single observation of the integrated free-tropospheric deuterium content in the tropics is approximately 25 permil^29^. Single-day averages over a ~2° latitude × 2° longitude area have an uncertainty of ~8 permil. The uncertainties in the data are somewhat correlated primarily due to the effects of temperature on the deuterium content retrieval^60^. However, further averaging is possible at monthly to seasonal time scales such that we expect the accuracy of averaged values at monthly time scales to be better than ~4 permil. This is because temperature acts in an almost random manner over monthly time scales.

As discussed in the next sub-section and demonstrated in previous studies^21,61^, the a priori constraint and averaging kernel must be applied to model-simulated atmospheric fields before comparison to the AIRS data. However, we find there is no observable impact on our conclusions from this linear operation because the sensitivity of the AIRS measurement is sufficient to resolve the tropospheric deuterium content. The AIRS deuterium content retrievals shown here infer the HDO/H_2_O ratio while also retrieving interfering effects such as cloud optical depth, atmospheric and surface temperature, and other trace gases that radiatively absorb and emit in the same 8 μm band. Cloud interference is therefore accounted for in the measurement. Nevertheless, because low sensitivity measurements are not used in this analysis, and because the effect of clouds in the middle and upper troposphere is to reduce the sensitivity of the measurement, the data used in this investigation may still have a clear sky bias.

To evaluate this possible bias, we use large-eddy simulations (LES) enabled with isotopic physic^55,62^. We consider two simulations of radiative-convective equilibrium: one without any large-scale vertical velocity and one with large-scale ascent, with the large-scale vertical velocity profile peaking at 500 hPa with a value of −60 hPa d^−1^. The simulations are run with a horizontal resolution of 750 m, which allows us to explicitly simulate convective clouds and their associated updrafts and downdrafts^55^. Ten snapshots of the simulations, corresponding to the last 10 days of the simulation, are analyzed here.

To emulate what the AIRS satellite would sample if it was flying in the LES atmosphere, we randomly sample 49 locations in each of the 10 snapshots. We calculate the average cloud water path in a 15 × 15 km pixel around each of these locations, corresponding to the AIRS footprint. If the cloud water path is higher than a threshold, the pixel is discarded as cloudy. Otherwise, it is considered clear-sky, and we calculate the average humidity and δ*D* profiles over the pixel. Finally, we average the humidity and δ*D* profiles over all the clear-sky pixels. We compare these profiles to the domain-mean values (Fig. S1 in Supplementary).

We find that the humidity and δ*D* profiles averaged over clear-sky pixels differ from those in the domain-mean by less than a few percentages and permil, respectively. This is the case even in the strong convective conditions that are simulated in the case with large-scale ascent. When looking at δ*D*_004, the difference is even smaller: the difference is only 0.04 permil and 0.7 permil for the case without and with large-scale ascent, respectively. Therefore, we can safely conclude that the effect of the clear-sky sampling bias can be neglected, likely because humidity and δ*D* vary at spatial horizontal scales that are much larger than the size of a cloud, due to very strong mixing between the clouds and their environment^63^.

### iCAM description and model-based simulations of deuterium content

We use the isotope-enabled Community Atmospheric Model Version 5 (herein iCAM), which includes isotopic physics routines and is able to simulate the modern distribution of water isotopologues in vapor and precipitation^64^. Comparison between iCAM δ*D* simulations^65^ and SCIAMACHY δ*D* observations^18^ in the tropics indicate a depleted bias, which could be associated with the difference between the temporal or spatial sampling of SCIAMACHY and the spatiotemporal resolution of the iCAM simulations. Nevertheless, iCAM is capable of simulating the spatial gradients of the isotopic distribution in the atmosphere, with fidelity^65^, and thus can be used for quantifying uncertainties in ET-P estimates based on δ*D*_004. In this study, we use the SST during 2002–2017 to drive iCAM at the 1.9° latitude ×2.5° longitude spatial resolution. We archive the monthly global output and perform analyses over the Amazon.

For simulating δ*D*_004 with the model, the mean AIRS averaging kernel and a priori constraint for the corresponding time periods, latitude, and longitude are applied to the HDO/H_2_O ratio from iCAM to account for the regularization and vertical resolution of the AIRS instrument. However, we find that because the AIRS sensitivity is sufficient to resolve the free troposphere, the application of the averaging kernel and a priori do not substantively change the vertical profile of iCAM δ*D*^29^.

### Quantifying the spatial representativeness of the deuterium-based ET-P proxy beyond the Amazon

One way to evaluate how well δ*D*_004 can quantify ET-P in the tropics is to quantify the correlations between δ*D*_004 and ET-P in one small region (e.g., a 2° × 2° grid cell) and between δ*D*_004 in this region and ET-P in other regions. This allows us to determine if the observed δ*D*_004 is representative of ET-P over that region and also to provide a measure of the spatial resolution of the ET-P estimate. We use δ*D*_004 from AIRS and ET-P obtained from PT-JPL ET and the mean precipitation of GPCP, TRMM, PERSIANN, and CRU as well as the δ*D*_004 and ET-P from iCAM. Here, we choose three ~2° × 2° regions in river basins 10 and 6 and in Venezuela, representing relatively wet (basin 10 and Venezuela) and dry (basin 6) areas of Amazonia. This analysis shows that the largest correlations between δ*D*_004 and ET-P from remote sensing are in the wet tropics (Fig. S3), whereas δ*D*_004 in the dry tropics is mostly correlated with ET-P in the wet tropics (Fig. S5). In other words, the highest correlation coefficient in Fig. S5 is not in the selected ~2° × 2° region, but in the regions north of 16°S and 61°W, likely because of the transport of air from the wet tropics into the dry tropics. The correlations become negative in the Northern Hemisphere (Figs. S3 and S5). Simulations from iCAM suggest the same conclusion, even though there are spatial pattern differences of δ*D*_004, ET, and precipitation between iCAM and observations. We will investigate in a subsequent manuscript if the use of deuterium in the boundary layer better represents nearby variations in ET-P in the dry tropics, which we might expect as previous studies suggest a strong relationship of continental recycling with near-surface deuterium content of water vapor^21^. Based on this test, we suggest caution in interpreting variations in δ*D*_004 with respect to ET-P in the mountain regions or in the dry tropics. The new deuterium proxy can at least resolve ET-P in the northern part of South America (e.g., the Venezuela/Guyana region; Fig. S4) as well as in the eastern, western, and southwestern part of the Amazon, as indicated by the skill in which the deuterium proxy can fit ET-P in these regions. The deuterium proxy can quantify ET-P in the south but additional analysis is needed to determine if this proxy has more information content than current approaches. A more refined estimate with finer spatial resolution could be quantified using the AIRS averaging kernel matrix^66^, but this is beyond the scope of this paper.

### Quantifying the uncertainty of ET-P with remote sensing observations and atmosphere models

We quantify the spatial accuracy of the deuterium-based ET-P estimates through comparison with different remote sensing products, re-analysis, TWS and discharge data, and iCAM. The GRACE TWS uncertainty is ~25 mm for an 800 km averaging radius^10^. To retain the accuracy of GRACE TWS and quantify the ET-P uncertainty at the same time, we, therefore, group the 14 river basins of Fig. 3b into five groups: Group 1 (basins 3, 10, and 12), Group 2 (basins 1, 13, and 14), Group 3 (basins 5, 8, and 9), Group 4 (basins 4, 6, and 11), and Group 5 (basins 2 and 7). For each group, we quantify the regression coefficient and its standard error, intercept, correlation coefficient, and the RMS error in the fit (mm month^−1^) between ET-P and δ*D* 004 from the different model and data sources. All comparisons are carried out using monthly data or monthly model output (Tables 1 and 2).

For each ET-P calculation in Table S2, we calculate the RMS error in the fit between an ET-P calculation and a corresponding ET-P estimation based on the ΑΙRS δ*D*_004 versus ET-P relationship. The different precipitation products are discussed in the previous section of “Methods”. It is noted that the ET obtained from Priestley-Taylor algorithm^46^ and from ERA-Interim suggest similar daily ET values (i.e., ~3.5 and ~3.2 mm day^−1^, respectively) and similar deviations from the mean of the reference datasets over the Amazon^67^. Here, the “reference datasets” refers to a combination of observational-based ET estimates, ET products from land surface model output, and ET from atmospheric reanalyses^67^. We also estimate the ET based on the fifth generation ECMWF re-analysis (ERA5) by using the latent heat flux (J m^−2^ day^−1^) from ERA5, which is then converted to ET (mm month^−1^; herein ERAET). Thus, we have nine distinct estimates of ET-P based on different combinations (Table S2), and calculate the RMS error in the fit between these nine ET-P and ET-P estimates based on AIRS δ*D*_004. By using the de-seasonalized time series in Table S2, we also quantify the RMS error in the fit based on the IAV of ET-P and AIRS δ*D*_004 in Table S3. Here, de-seasonalized variables are obtained by subtracting the multi-year mean of each month from corresponding month of the time series. In addition, the RMS error in the fit between the ET-P estimates from ET-P–δ*D*_004 relationship and ET-P simulation are calculated with iCAM output (Table S4). These different comparisons are used to demonstrate the uncertainties in the seasonality and IAV of the δ*D*_004 based ET-P estimates.

## Supplementary information

Supplementary Information

## Data Availability

The source data used to generate all plots and graphs and the plotting scripts are provided at https://zenodo.org/record/6404457#.YkaWWm7MJEI. Due to the large size of the raw data and iCAM output, these files were not deposited in a public repository, but are available from the corresponding authors on reasonable request.
