# Supplementary material for: Amazonian terrestrial water balance inferred from satellite-observed water vapor isotopes
Source: Nat Commun. 2022 May 13;13:2686. doi: 10.1038/s41467-022-30317-4 (PMC9106687; doi:10.1038/s41467-022-30317-4)
Supplement: Supplementary file 1 — Supplementary Information [file 41467_2022_30317_MOESM1_ESM.pdf]

Supplemental information for:

Amazonian terrestrial water balance inferred from satellite-observed water vapor isotopes

1. Joint Institute for Regional Earth System Science and Engineering, University of California, Los Angeles
2. Pacific Northwest National Laboratory, 902 Battelle Blvd, Richland, WA 99354
3. Jet Propulsion Laboratory, California Institute of Technology
4. National Center for Atmospheric Research
5. University of Auckland
6. Laboratoire de Météorologie Dynamique
7. University of California, Los Angeles
8. California Institute of Technology, Pasadena,
9. Schmid College of Science and Technology, Chapman University, 1 University Drive, Orange, CA, 92866, USA

* Corresponding Authors: Mingjie Shi and John Worden

Mingjie Shi: mingjie.shi@pnnl.gov

John Worden: john.r.worden@jpl.nasa.gov

Table of Contents

***Figure S1* 3**

***Figure S2.* 4**

***Figure S3* 4**

***Figure S4.* 5**

***Figure S5.* 6**

***Table S1*.......... 7**

***Table S2*.......... 7**

***Table S3*.......... 8**

***Table S4*.......... 8**

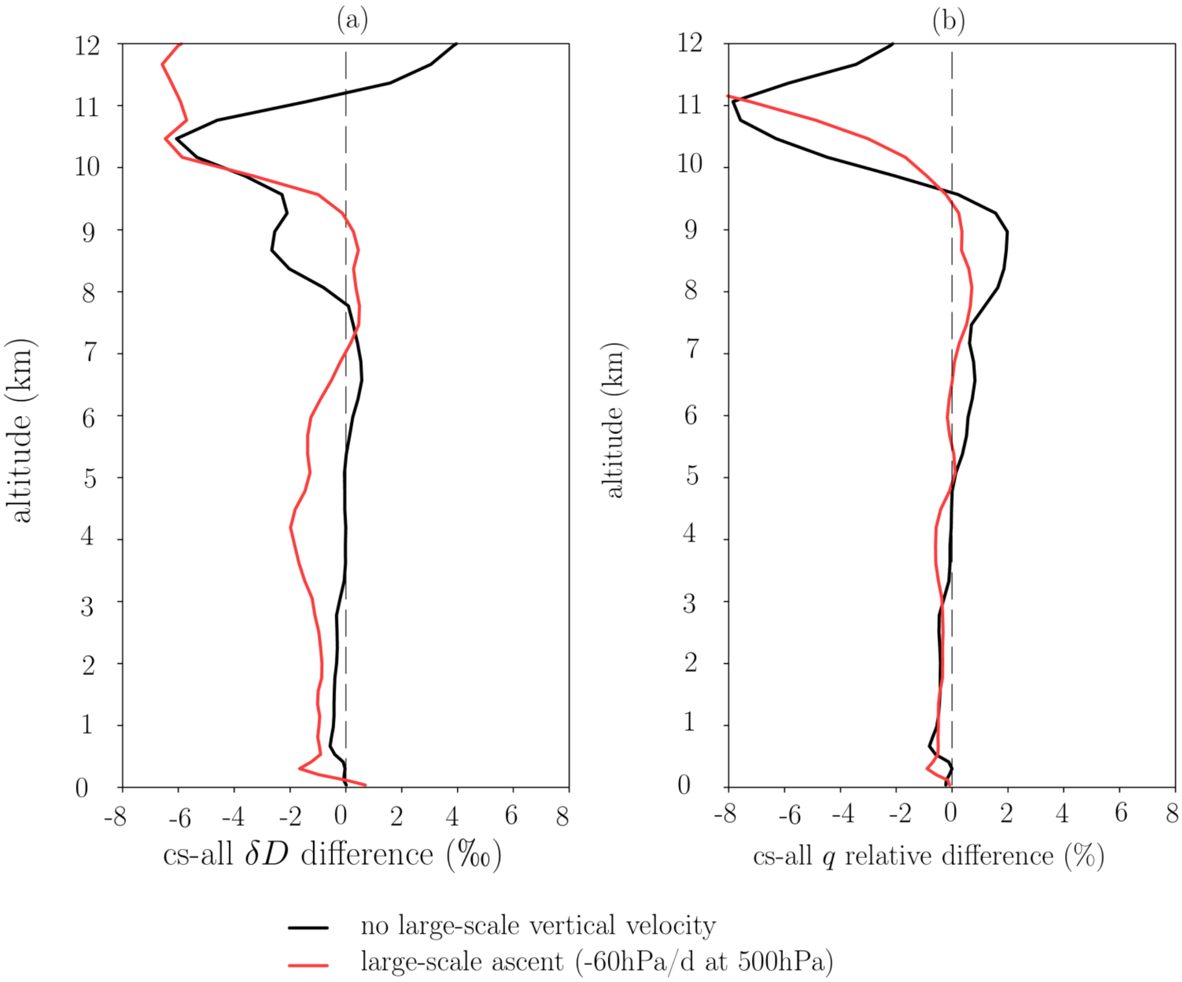

Figure S1. a) Water vapor δD difference between the average over randomly chosen clear-sky pixels and the domain-mean, for two simulations : one without any large-scale vertical velocity (black) and one with large-scale ascent (red). (b) Same for the relative difference in specific humidity. For the case without any large-scale vertical velocity, the fraction of pixels that were diagnosed as clear sky is 22% and the average is calculated over 107 pixels. For the case with large-scale ascent, the fraction of pixels that were diagnosed clear sky is 10 % and the average is calculated over 50 pixels.

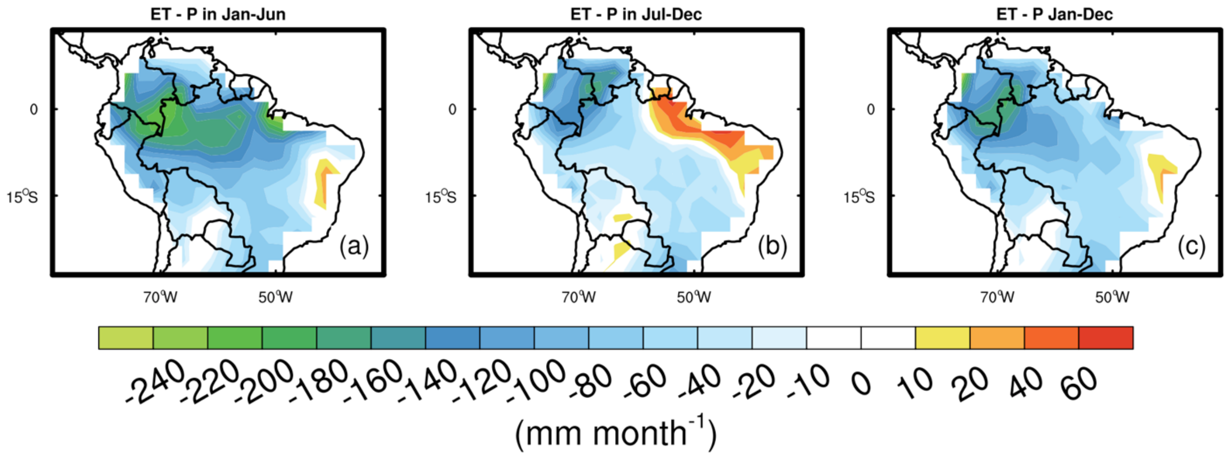

Figure S2. PT-JPL ET and GPCP precipitation suggested ET-P (mm month^{-1}) during (a) January–June, (b) July–December, and (c) January–December over the Amazon.

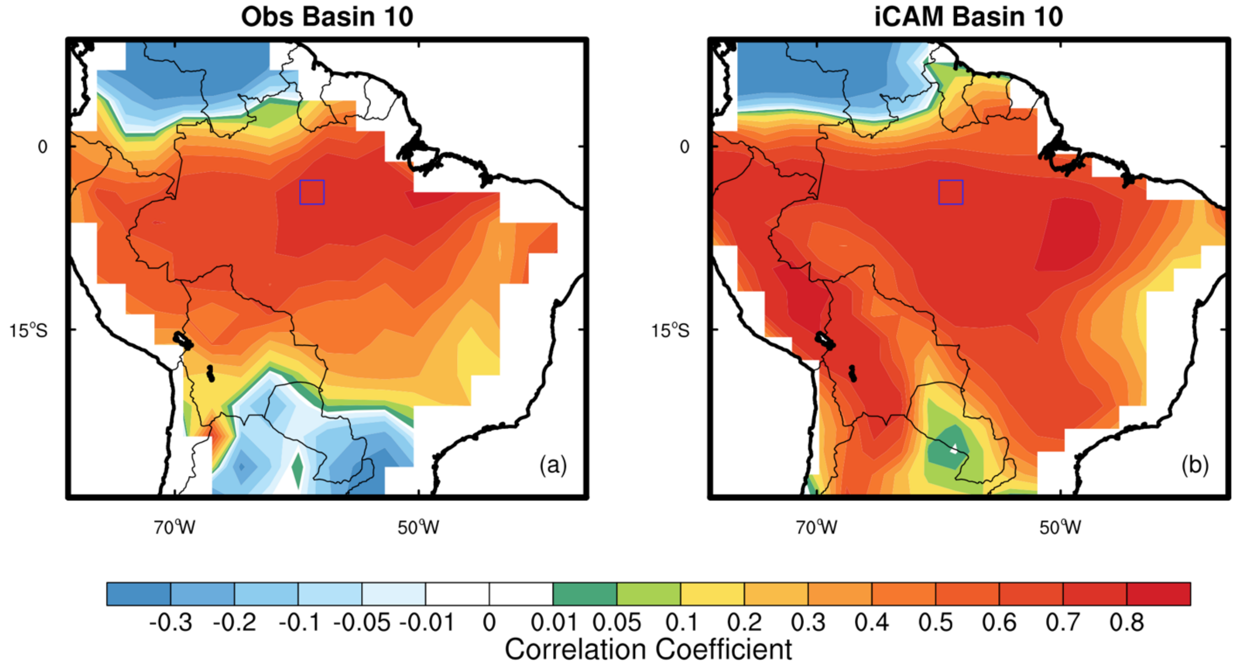

Figure S3. The spatial pattern of the correlation coefficient (a) between δD_004 from AIRS and ET-P obtained from PT-JPL ET and the mean precipitation of GPCP, TRMM, PERSIANN and CRU, and (b) between δD_004 and ET-P from iCAM during 2013–2015. The blue box represents the selected ~2° ×2° region, which is in Basin 10 and the center is at 4°S and 59°W. The correlations between δD_004 in that region and ET-P in other regions of the Amazon are calculated.

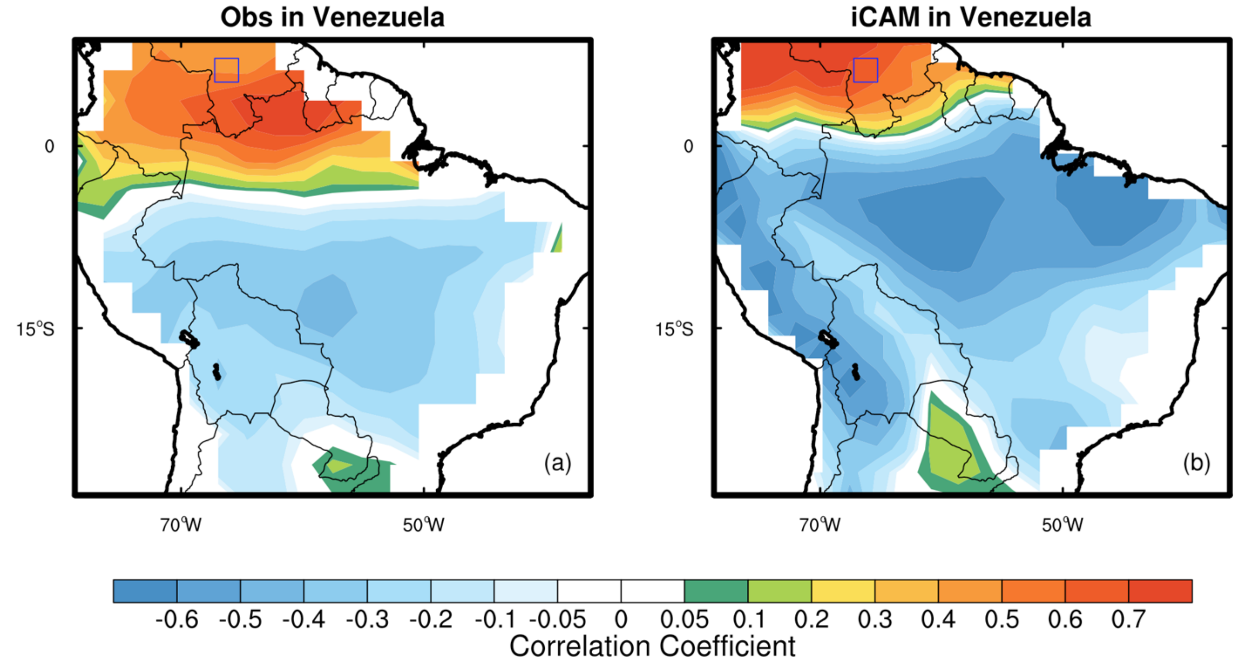

Figure S4. The spatial pattern of the correlation coefficient (a) between δD_004 from AIRS and ET-P obtained from PT-JPL ET and the mean precipitation of GPCP, TRMM, PERSIANN and CRU, and (b) between δD_004 and ET-P from iCAM during 2013–2015. The blue box represents the selected ~2° ×2° region, which is in Venezuela and the center is at 6°S and 67°W. The correlations between δD_004 in that region and ET-P in other regions of the Amazon are calculated.

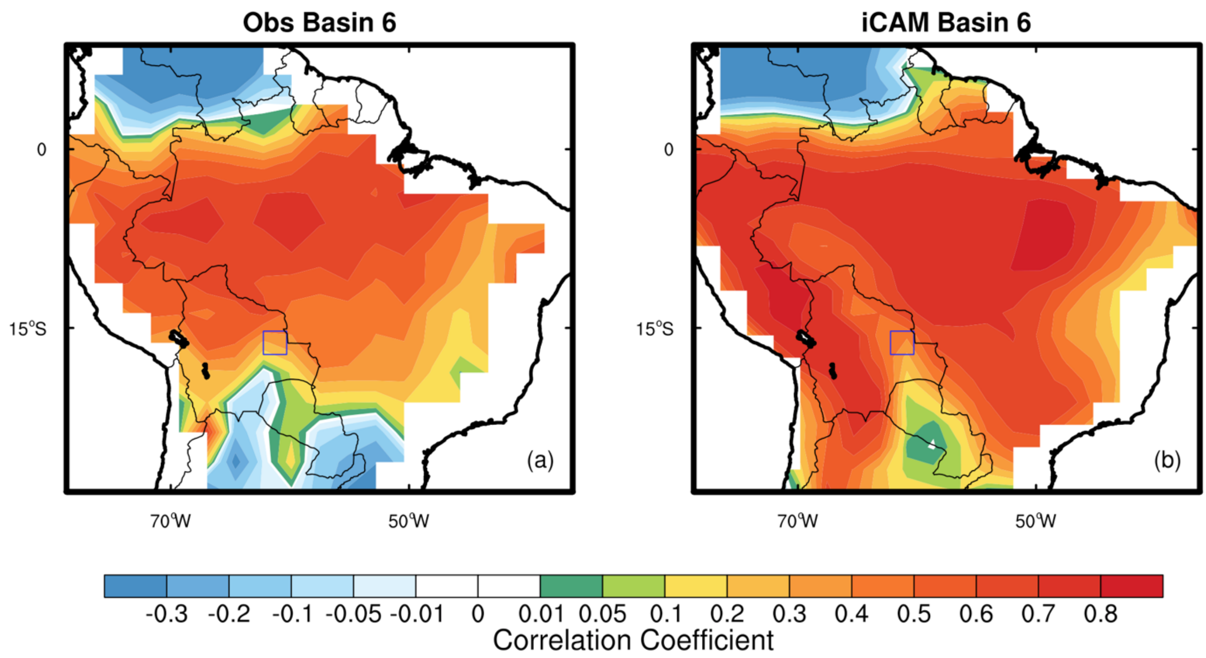

Figure S5. The spatial pattern of the correlation coefficient (a) between δD_004 from AIRS and ET-P obtained from PT-JPL ET and the mean precipitation of GPCP, TRMM, PERSIANN and CRU, and (b) between δD_004 and ET-P from iCAM during 2013–2015. The blue box represents the selected ~2° ×2° region, which is in Basin 6 and the center is at 16°N and 61°W. The correlations between δD_004 in that region and ET-P in other regions of the Amazon are calculated.

Tables.

Table S1. The basin description of the 14 Amazon river basins in Figure 3b.

**Basin index**	**Basin names**	**Basin area (km^2)**
1	Acanaui	250800
2	Altamira	469200
3	Caracarai	131100
4	FzVistaAlegre	338800
5	Gaviao	164700
6	Guayaramerin	640400
7	Itaituba	461500
8	Labrea	229600
9	Manacapuru	438900
10	Obidos	747800
11	PortoVelho	378000
12	Serrinha	290200
13	StoAntonioDoIca	264500
14	Tabatinga	883200

Table S2. Root-Mean Square (RMS) error in the fit (mm month^{-1}) between monthly ET-P estimations from AIRS δD_004 and different ET and precipitation combinations (ET-P) in the same 5 basin groups of Table 1. The calculations use monthly records across the study time period of 2003–2015. One of the ET-P products is derived from TWS/discharge, and the other eight ET-P combinations are listed below.

Group number	TWS/ discharge	1	2	3	4	5	6	7	8
1	43.11	57.98	81.58	54.57	53.33	54.25	43.88	51.53	50.06
2	35.86	30.97	90.81	34.32	39.01	31.33	30.84	36.71	38.89
3	45.86	44.24	100.92	50.02	47.73	48.52	53.37	55.43	52.07
4	52.89	51.56	82.98	54.49	51.39	54.51	54.97	57.41	53.43
5	77.85	67.89	94.31	85.22	72.57	71.78	78.81	89.54	76.53

1 = ERAET-GPCP, 2 = ERAET-TRMM, 3 = ERAET-PENNSIANN, 4 = ERAET-CRU, 5 = JPLET-GPCP, 6 = JPLET -TRMM, 7 = JPLET -PENNSIANN, 8 = JPLET-CRU

Table S3. RMS error in the fit (mm month^{-1}) based on interannual variability (IAV) of monthly ET-P estimations from AIRS δD_004 and that of different ET and precipitation combinations (one of the ET-P products is derived from GRACE TWS and river discharge) for the same 5 basin groups of Table 1. The calculations use deseasonalized monthly records across the study time period of 2003–2015. Similar to Table S1, de-seasonalized ET-P and δD_004 are used for the calculation in this table.

Group number	TWS/ discharge	1	2	3	4	5	6	7	8
1	37.82	35.95	70.07	38.46	42.24	36.69	35.75	39.70	42.81
2	26.27	27.52	75.05	29.43	37.73	25.98	25.34	30.37	36.38
3	36.89	31.57	80.54	29.55	34.89	31.58	31.77	29.64	34.74
4	18.55	25.75	75.06	23.10	29.87	25.82	26.88	23.35	30.41
5	23.05	33.20	78.85	38.02	20.72	32.35	29.58	38.14	21.03

1 = ERAET-GPCP, 2 = ERAET-TRMM, 3 = ERAET-PENNSIANN, 4 = ERAET-CRU, 5 = JPLET-GPCP, 6 = JPLET -TRMM, 7 = JPLET -PENNSIANN, 8 = JPLET-CRU.

Table S4. iCAM suggested RMS error the in fit (mm month^{-1}) between monthly ET-P and monthly ET-P estimated with δD_004 for the same 5 basin groups of Table 1. Here, we use monthly iCAM output during 2003–2015.

Group number	δD_004 vs ET-P	δD_004 vs ET-P IAV
1	32.60	23.40
2	32.99	14.79
3	31.99	16.54
4	36.57	17.57
5	41.41	19.17
